# “It’s Not Only Attention We Need”: Systematic Review of Large Language Models in Mental Health Care

**DOI:** 10.2196/78410

**Published:** 2025-11-04

**Authors:** Andreas Bucher, Sarah Egger, Inna Vashkite, Wenyuan Wu, Gerhard Schwabe

**Affiliations:** 1 Department of Informatics University of Zurich Zurich Switzerland

**Keywords:** AI, GenAI, large language models, LLM, mental health, systematic review

## Abstract

**Background:**

Mental health care systems worldwide face critical challenges, including limited access, shortages of clinicians, and stigma-related barriers. In parallel, large language models (LLMs) have emerged as powerful tools capable of supporting therapeutic processes through natural language understanding and generation. While previous research has explored their potential, a comprehensive review assessing how LLMs are integrated into mental health care, particularly beyond technical feasibility, is still lacking.

**Objective:**

This systematic literature review investigates and conceptualizes the application of LLMs in mental health care by examining their technical implementation, design characteristics, and situational use across different touchpoints along the patient journey. It introduces a 3-layer morphological framework to structure and analyze how LLMs are applied, with the goal of informing future research and design for more effective mental health interventions.

**Methods:**

A systematic literature review was conducted across PubMed, IEEE Xplore, JMIR, ACM, and AIS databases, yielding 807 studies. After multiple evaluation steps, 55 studies were included. These were categorized and analyzed based on the patient journey, design elements, and underlying model characteristics.

**Results:**

Most studies assessed technical feasibility, whereas only a few examined the impact of LLMs on therapeutic outcomes. LLMs were used primarily for classification and text generation tasks, with limited evaluation of safety, hallucination risks, or reasoning capabilities. Design aspects, such as user roles, interaction modalities, and interface elements, were often underexplored, despite their significant influence on user experience. Furthermore, most applications focused on single-user contexts, overlooking opportunities for integrated care environments, such as artificial intelligence–blended therapy. The proposed 3-layer framework, which consists of the L1: LLM layer, L2: interface layer, and L3: situation layer, highlights critical design trade-offs and unmet needs in current research.

**Conclusions:**

LLMs hold promise for enhancing accessibility, personalization, and efficiency in mental health care. However, current implementations often overlook essential design and contextual factors that influence real-world adoption and outcomes. The review underscores that the self-attention mechanism, a key component of LLMs, alone is not sufficient. Future research must go beyond technical feasibility to explore integrated care models, user experience, and longitudinal treatment outcomes to responsibly embed LLMs into mental health care ecosystems.

## Introduction

### Mental Health Care and Digital Technologies

With nearly a billion individuals facing mental health conditions, the importance of mental health care has become prominent [1-3]. However, most people do not have access to mental health care, as there is a clear lack of clinical workforce in lower-income [4] and even in developed countries [5]. Additionally, the financial burden and existing stigma around mental health care often discourage individuals from seeking help [6]. Nonetheless, even when having access to mental health care services, patients are often confronted with inefficiencies, such as long waiting times for initial consultations or inadequate referrals to specialists. These issues impair the effectiveness of the therapy and leave patients without adequate support. Furthermore, the critical shortage of therapists and medical professionals and the inefficiencies of the mental health care system place a heavy burden on therapists and medical professionals. This raises job-related stress, resulting in lower job satisfaction and higher burnout rates [7]. Additionally, many therapists are emotionally affected [8,9]. This makes it increasingly difficult for them to maintain a neutral therapeutic stance and deliver effective treatment.

Hence, new digital approaches have been explored to support therapists and patients along the patient journey. Therein, an effective and efficient way to provide therapeutic support to a large number of geographically dispersed patients is online psychotherapy (OP). OP bridges and reduces temporal and geographic restrictions of psychotherapy by using messenger applications or tele- and videoconferencing solutions [10]. Various studies found that OP is a reliable and effective method for multiple diagnoses and behavioral health treatments, including major depressive disorder, schizophrenia, bipolar disorder, and attention disorder [11,12]. Although therapists and patients connect and interact online through text, audio, or video communication, they are still able to form a therapeutic alliance similar to in-person meetings [13]. It establishes a personal bond between patients and therapists, satisfying their need for relatedness, which results in higher trust and openness, and promotes a sense of purpose and effectiveness [14,15]. However, despite the positive effects of OP, it still requires the involvement of a therapist or medical professional and, thus, does not solve their shortage and availability issue.

In contrast, technological advancements have led to a broad proliferation of self-guided therapy applications such as Headspace [16], Worry Watch [17], or Calm [18]. In self-guided therapy, patients rely on technology, such as mobile apps or websites, to independently manage their mental health [19,20]. For example, Firth et al [21] found a decrease in depressive symptoms for mild depression and high app adherence for treating symptoms of schizophrenia with mobile apps. Additionally, Ly et al [22] observed a reduction in total anxiety when using mobile apps for therapy. Such applications for self-guided therapy, thus, lower entry barriers, offer treatment support, and increase mental strength. However, many applications used within self-guided therapy base their response on predefined rules, restricting user input and interactions [23]. Some applications, such as Replika, have been effective in forming personal bonds between users and the application, even to the extent of developing addictions or parasocial relationships, which call for more socio-affective alignment with artificial intelligence (AI) [24-27]. However, many self-guided therapy applications are typically not as effective in creating a bond and therapeutic alliance as human therapists are [28,29]. Hence, there is a high number of users who do not adhere to the exercises and quit treatment early. Against this background, the rapid pace of progress in large language models (LLMs) offers new opportunities for more adaptive and naturalistic self-guided therapy. LLM-based conversational agents (CAs), which are capable of simulating human-like dialogue, have quickly surpassed earlier rule-based systems and continue to evolve at extraordinary speed, with new models and capabilities emerging in rapid succession [30].

### LLMs

With rising awareness of mental health care, the interest in using technology, including language models, to support and bridge gaps in mental health care has also increased. With the paper *Attention Is All You Need* [31], the world was introduced to the self-attention mechanism, laying the foundation for later developments of the transformer architecture and LLMs [32]. Our title deliberately echoes this landmark work. While *Attention Is All You Need* [31] established the technical foundation for modern LLMs, our review asks whether attention alone is sufficient when applying these models in the sensitive domain of mental health care. This question seems important given the rapidly expanding technical capabilities of LLMs.

LLMs, which focus on scaling model and data size, have demonstrated strong task-solving capabilities within various real-world applications [33]. Central to this progress is the self-attention mechanism, which enables models to capture dependencies within sequences, such as the semantic meaning of words, and to use these representations for downstream tasks. Today, LLM capabilities are often grouped into discriminative tasks (eg, classification or prediction), generative tasks (eg, producing new content or responses), and, more recently, reasoning tasks supported by prompting strategies that enable stepwise inference.

Earlier transformer-based models, such as Bidirectional Encoder Representations from Transformers (BERT) or RoBERTa, have demonstrated groundbreaking performance and pushed the state of the art for many benchmarks in natural language understanding, especially in classification and translation tasks [34,35]. The introduction of the first autoregressive models, such as OpenAI’s Generative Pretrained Transformer (GPT) and GPT-2, further extended the scope of LLMs to natural language generation by predicting token by token. However, it was not until the publication of ChatGPT that the general public became aware of this technology and learned about its potential. Modern LLMs, such as OpenAI’s GPT-4 [36] or Meta’s LLaMa 2 [37], are nowadays capable of processing various input modalities to generate multimodal output, including textual, auditive, or visual results. Nonetheless, despite their capabilities in generating human-like texts and highly realistic images or videos, these models still fall short in logical reasoning or in drawing connections in the underlying input data beyond probabilistic pattern matching [38].

Chain-of-thought (CoT) prompting is one strategy to enhance the reasoning capabilities of LLMs by breaking down the initial problem into a series of intermediate reasoning steps before arriving at a conclusion [39]. Furthermore, enhancements in model training and architecture, such as training on CoT data, have led to LLMs being specifically designed for multistep logical reasoning, such as OpenAI’s o1 model [40]. Despite these enhancements, LLMs remain vulnerable to hallucinations and misinformation. In response, researchers have explored countermeasures, such as techniques to flag potential hallucinations [41] or explainable AI (XAI) methods [39,42-44] to portray a realistic picture of what AI can and cannot do. Additionally, beyond technical solutions, fostering AI literacy among users is equally important. A higher level of AI literacy enhances comprehension of LLM behavior and promotes more effective human-AI collaboration [45,46].

Nowadays, LLMs are widely available and can be accessed in a variety of ways, such as closed-source (CS), open-source (OS), or open-weight (OW) [47]. OS and OW models can both be deployed on an organization’s own infrastructure and servers, that is, on-premises or in a private cloud, allowing more control over inference and fine-tuning. However, they differ in terms of access to the underlying model architecture and transparency. OS models provide access to both the model architecture and weights, which enables full customizability. OW models, in contrast, typically only provide pretrained model weights, allowing fine-tuning or inference when paired with compatible OS implementations of the architecture. CS models are hosted on external servers and are only accessible through application programming interfaces (APIs), commonly operating on a pay-as-you-go basis. Consequently, the choice of model type has far-reaching implications, not only for data privacy and sovereignty but also for the degree of control an organization has over model customization, including techniques such as fine-tuning, retrieval-augmented generation (RAG), or advanced prompting strategies.

### AI in Mental Health Care

Following rapid technical advancements and the consistent release of newer and more capable LLMs, their proliferation in people’s private and professional lives has rapidly increased. These developments not only found their way into the broader medical domain [48-51] but also into mental health care, as the number of applications for online therapy has continuously risen. Traditionally, the primary approach for delivering mental health care involves dialogues between patients and therapists. Therein, reliance on the conversational context is central for formulating a diagnosis, suggesting treatment paths, and delivering therapeutic measures. Given the dialog capabilities of recent LLMs, they appear suited for mental health care and promise to make mental health care more broadly accessible.

However, integrating conversational user interfaces (CUIs) in mental health care is nothing novel. ELIZA, one of the first chatbots, demonstrated already in the 1960s the capabilities of software-based agents in psychotherapy [52]. Ever since, CUIs have become widely used in mental health care and have evolved to be an essential technology in supporting clinicians and health care administrators in various mental health–related tasks. For example, Bickmore et al [53] showed that chatbots for psychiatric applications are able to offer assistance to individuals who would otherwise not seek help due to stigma or cost. Furthermore, chatbots are effective in identifying specific mental conditions [54], building emotional support [55], or supporting interventions [56]. Since the introduction of the self-attention mechanism and the first transformer-based architectures [31,32], natural language processing and the development of CUIs have increasingly shifted toward LLMs. These models prioritize scaling in terms of parameters and data, enabling strong task-solving capabilities across various real-world applications [33,57]. Their effectiveness has also fueled growing research interest in applying LLMs to mental health care, which has led to numerous studies exploring their potential in this domain [58].

Several reviews have since consolidated findings from technical research and empirical studies involving patients, therapists, and other users of LLM-based chatbots and digital agents for mental health care. Most of these reviews highlight the potential applications of LLMs in mental health care while also addressing their challenges and limitations. For example, Guo et al [58] found that many studies focus on using LLMs to detect mental health conditions and suicide ideation in text. They also examined how LLMs are integrated into mental health CAs to provide mental health support and interventions. Similarly, Lawrence et al [59] discussed how LLM applications can be used for mental health education, assessment, and interventions. They also mentioned several limitations and challenges that accompany the implementation of LLMs in mental health care, especially for more complex and high-risk scenarios, calling for human intervention.

Although these reviews underscore the effectiveness of LLMs in mental health care, they primarily concentrate on the patient perspective. They do not extensively analyze broader contextual factors, such as the specific tasks LLMs fulfill, their adaptation to context-specific information and boundaries, interaction modalities, or the integration of social cues in LLM-based applications. However, when designing and implementing such applications, it is essential to consider multiple design dimensions that influence their capabilities, usability, and acceptance. In the development of CAs, design elements, such as role, gender, visual representation, voice, or nonverbal cues [60-65], greatly determine the adoption and impact of CAs. Similar can be expected for LLM-based applications in mental health care. Despite bringing new attention, both in technical terms and also in the broader societal, economic, and scientific context, to mental health care applications, we believe that the integration of LLMs will not solve all challenges. Instead, while the LLM serves as the core engine powering these applications, it is the surrounding design choices, such as their focus on specific tasks, their interaction modalities, the integration of social cues, or their positioning within the patient journey, that ultimately shape usability and user experience.

Hence, this systematic review raises and critically approaches the question, “Is attention all we need?” Therein, it explores the application of LLMs in mental health care from a broader perspective, covering various design choices, and looks well beyond the self-attention mechanism that powers today’s LLMs as introduced in the seminal paper *Attention Is All You Need* [31]. This systematic review was conducted to derive insights for future research and design from both a scientific and practical point of view to develop safer and more responsible LLMs for mental health care. Therein, the review uncovers interesting insights concerning the design and impact of LLMs in mental health care and derives a design taxonomy for analyzing previous applications, but also to guide future research and development.

## Methods

### Search Approach and Strategies

To identify, summarize, and categorize the use of LLMs in mental health care, as well as their design and impact, we conducted a systematic literature review. As the goal of this study is to conceptualize existing approaches of LLMs in mental health care and to understand complex sociotechnical phenomena, we followed the approach of vom Brocke et al [66]. This approach is widely recognized in information systems research and emphasizes concept-centric analysis, iterative literature saturation, and alignment with sociotechnical research aims [67-69]. An adapted PRISMA (Preferred Reporting Items Systematic Reviews and Meta-Analyses) checklist for the conceptual, theory-driven systematic literature review can be found in Multimedia Appendix 1.

Therein, we identified, evaluated, and synthesized existing research on LLMs in mental health care [66]. To identify relevant studies, we relied on keyword search. For this, we constructed the keywords of 2 parts—one including keywords related to LLMs and one containing keywords related to mental health care and different touchpoints along the patient journey. The keywords were defined as follows: (Large Language Models) OR (LLM applications) AND (mental healthcare) OR (psychoeducation) OR (consultation) OR (psychotherapy), as well as (chatbot) AND (mental healthcare) OR (psychoeducation) OR (consultation) OR (psychotherapy). The keyword “chatbot” was added, as many implementations of LLMs are chatbots, and it could be expected that many studies focused on specific chatbots.

In the identification, the search terms were used on the platforms PubMed, IEEE Xplore, JMIR, ACM, and AIS with April 30, 2025, as the cutoff date. Focusing on these platforms served as quality assurance, as only peer-reviewed studies were selected for the review process. Although gray literature and articles published on preprint servers could provide timely insights in this rapidly evolving field, they were explicitly excluded to ensure that only peer-reviewed and, thus, quality-assured studies formed the evidence base. This reduces the risk of including speculative or unstable findings. Overall, the search returned 807 papers.

After the identification, all papers were subject to rigorous screening, which included duplicate removal (n=114) and the screening of titles, keywords, and abstracts of the remaining 693 papers. This was done by creating a Python script. The script applied keyword searches to identify papers containing terms related to LLMs (LLM, Large Language Model, OpenAI, ChatGPT, Bard, Claude, GPT, BERT, T5, Megatron, NVIDIA, LLaMA, OPT, and MiniLM), and mental health (mental health, psychoeducation, psychotherapy, psychiatry, psychological, and psychiatric). Based on the keyword matches, the script generated 2 CSV files: one comprising articles where all specified keywords were present and another where at least one keyword was missing. Through this selection process, 217 papers were selected for detailed evaluation.

In the next step, the full-text articles (n=217) were assessed for eligibility. Therein, the abstracts were read and analyzed. In addition, these papers were then analyzed in more detail by checking what type of applications were involved and if any implementation of an LLM was mentioned. Through this process, another 174 papers were removed for various reasons. While most excluded papers focused on CAs (n=110), general AI applications (n=9), or other technological approaches (n=45) in mental health care, they did not mention the use of LLMs. Additionally, other excluded papers did not specifically focus on mental health care (n=10).

In the final step, the synthesis, the remaining papers (n=43) were categorized according to different patient journey touchpoints, different design elements, and the underlying LLM. At this step, additional papers (n=12) were identified and included in the review using back and forward search, resulting in a total of 55 included studies that were analyzed in detail in the synthesis. A summary of the reviewed articles (n=55) on LLMs in mental health care with their categorizations can be found in Multimedia Appendix 2.

**Figure 1:**
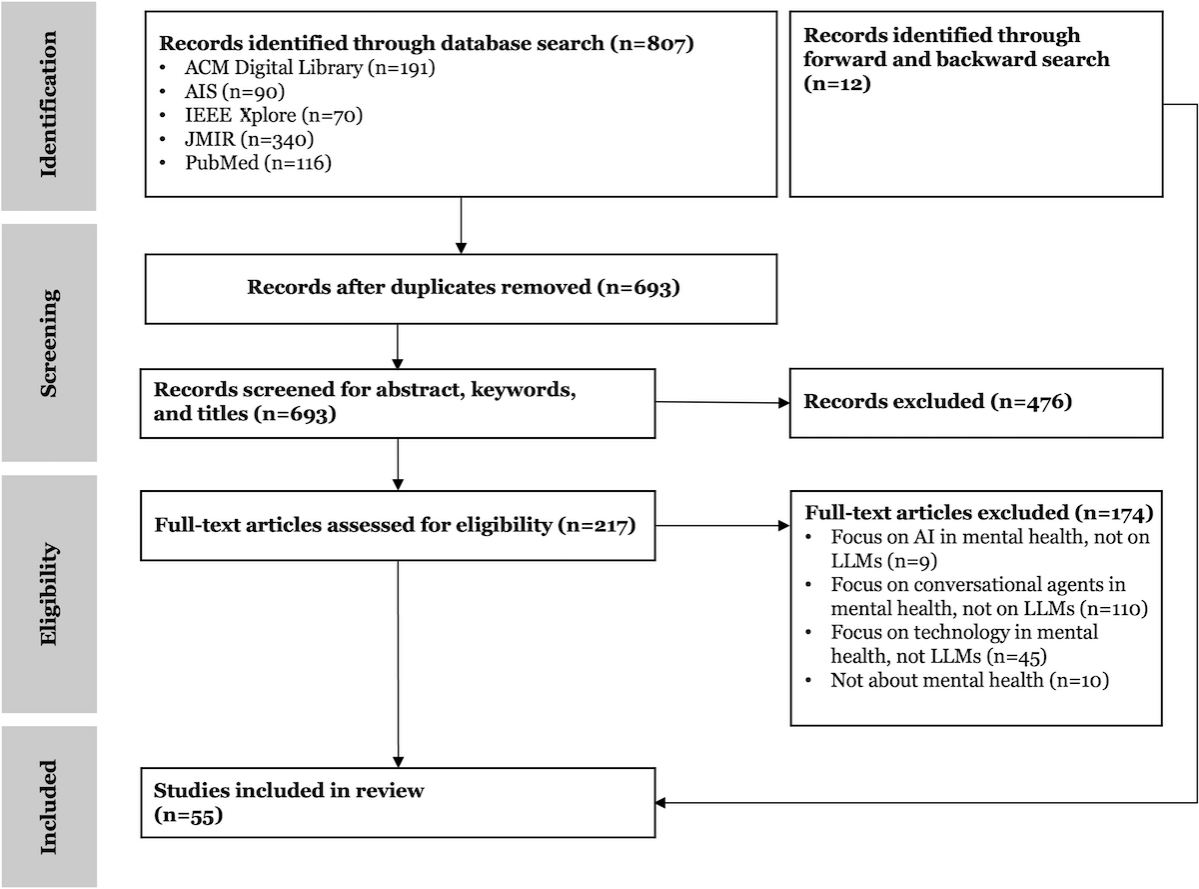
Process of the systematic literature review following vom Brocke et al [66]. AI: artificial intelligence; LLM: large language model.

### Information Extraction During Synthesis

The information extraction process during the final synthesis step was jointly conducted by 3 researchers. First, all identified papers were read in detail and notes taken about the model provenance, the usage of the LLM, and the broader design features of the application. Additionally, further papers were identified using backward and forward search during this activity. Second, a high-level coding scheme was developed after the first reading, covering 25 high-level codes, such as supported task type, therapeutic approach, step within the patient journey, used LLM, scope of the LLM, interaction modalities, primary and secondary user group, and even study type.

Based on this coding scheme, all papers of the final selection were then iteratively coded using top-down coding [70] in a third step. Because the coding categories were refined throughout this process, calculating interrater agreement statistics was not meaningful. Instead, a subset of the papers was independently coded by multiple researchers, and discrepancies were documented and resolved through discussion until consensus was reached. This iterative comparison and consensus-building ensured shared understanding of the codes and consistency in their application.

Additionally, as many papers did not explicitly mention which LLM they used or which dataset they relied on during fine-tuning, we attempted to find further information about their studies on the internet, for example, on GitHub or specific project websites. Finally, to ensure interrater reliability and to prevent subjectivity, the codes were grouped and discussed by the authors. In the last step, recurring themes, such as the supported therapeutic task, the user, or the model provenance, were analyzed in detail. The reasons for the full-text exclusion of 174 papers can be found in Multimedia Appendix 3.

## Results

### Overview of the Analyzed Studies

Figure 1 illustrates the systematic search process, which resulted in a final set of 55 papers included in this review. The review of the identified studies shows that most (37/55, 67%) studies have focused on the technical feasibility of using LLMs in mental health care, followed by meta-studies (14/55, 26%), such as reviews, editorials, opinion papers, or online surveys. Lastly, only a few studies (4/55, 7%) actually assessed the impact of LLMs on therapeutic measurements and treatment-related outcomes (eg, adherence; Table 1).

Various feasibility studies have demonstrated the technical capabilities of using LLMs in different contexts and tasks, for example, to detect specific mental issues, disorders, or emotional states; to train mental health experts; to provide recommendations; or to conduct specific exercises. It is to note that these studies have only evaluated the technical capabilities of LLMs, for example, the classification accuracy of detecting depression symptoms in written texts, and have not been tested with real patients. Only a few studies have evaluated the impact of LLMs on people with mental health issues, for example, to improve treatment adherence or to change emotions. However, even among these few articles that focused on the actual impact of LLMs in mental health care, only 2 studies included diagnosed patients in their evaluation. The last category of articles analyzed is meta-studies, including reviews, editorials, opinion papers, and online surveys concerning the use of LLMs for mental health care.

**Figure 2:**
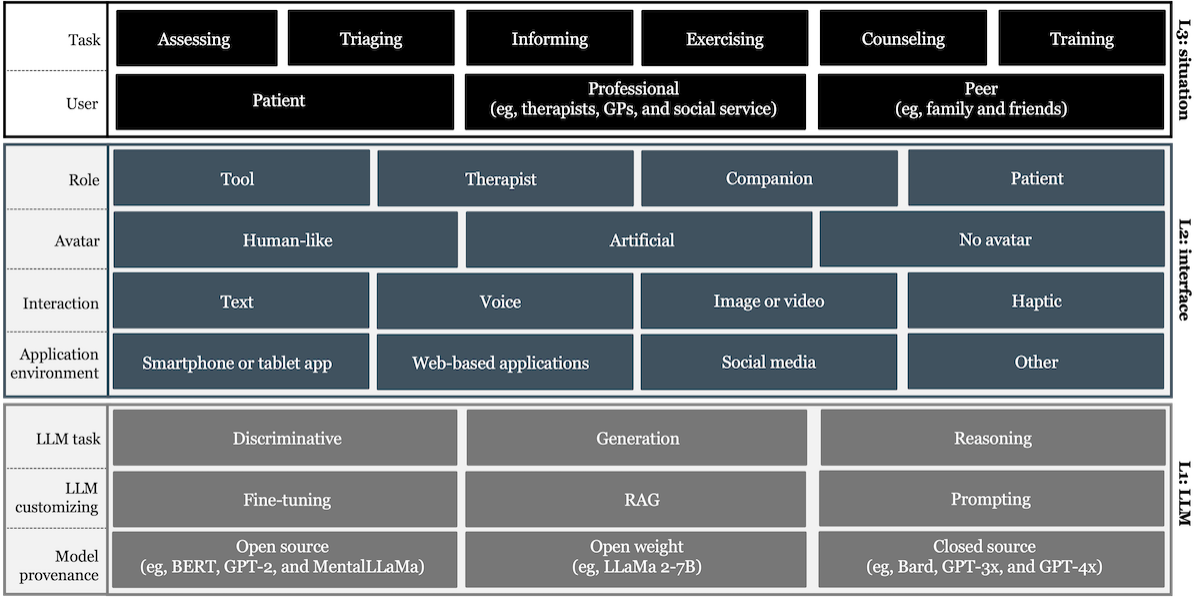
Morphological box concerning the integration of large language models (LLMs) in mental health care. BERT: Bidirectional Encoder Representations from Transformers; GP: general practitioner; RAG: retrieval-augmented generation.

Based on the analyzed literature, a morphological box (Figure 2) concerning the integration of LLMs in mental health care was derived to categorize the use of LLMs in mental health care. Therein, it comprises three distinct layers: (1) the LLM layer (L1), (2) the interface layer (L2), and (3) the situation layer (L3). These 3 layers help to characterize the use of LLMs along situational aspects and atomic design decisions. Furthermore, each layer entails multiple sublayers to further break down the layers into concrete aspects and design choices. All in all, 3 layers with a total of 9 sublayers were identified in the literature. In the following, each layer is described in detail.

**Table 1:** Categorization of analyzed studies.

Types of studies and their focus		Values, n (%)	Reference
**Feasibility and design studies**		37 (67)	
	Detecting mental issues, disorders, or emotional states	14 (25)	[71-84]
	Providing information and recommendations to patients, peers, and mental health professionals	7 (13)	[85-91]
	Conducting exercises with patients	5 (9)	[92-96]
	Training of mental health professionals	4 (7)	[97-100]
	Other (eg, summarization of counseling sessions, response assessment capabilities of LLMs^a^, etc)	7 (13)	[101-107]
**Effect studies**		4 (7)	
	Studies with diagnosed patients	2 (3.5)	[108,109]
	Studies with the general population	2 (3.5)	[110,111]
**Meta-studies**		14 (25)	
	Reviews, editorials, and opinion papers	11 (20)	[58,59,112-120]
	Online surveys on the general use of artificial intelligence agents for mental health care	3 (5)	[121-123]

^a^LLM: large language model.

### L1: LLM Characteristics as Foundation for New Forms of Mental Health Care

#### Overview

As the domain of mental health care is highly sensitive, particularly regarding data privacy, transparency, and control over LLM outputs, the choice of the underlying model type requires careful consideration. This includes not only the model’s architecture and the accessibility of its weights but also its adaptability to domain-specific requirements and the range of tasks it can perform. These considerations are described by the 3 sublayers: model provenance, LLM customizing, and LLM task.

#### Model Provenance and LLM Customizing

**Figure 3:**
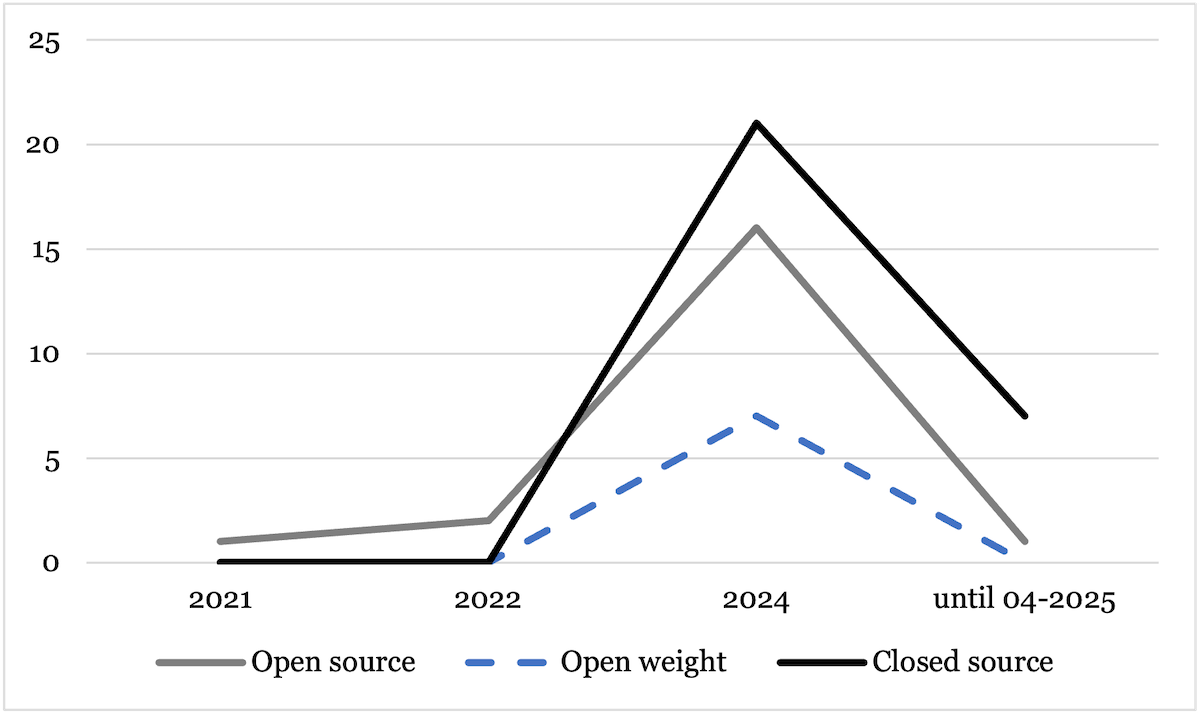
Model provenance in scientific research on large language models in mental health care over the years.

Research on LLMs in mental health care has rapidly increased over the last couple of years, as can be seen in Figure 3, which describes the model provenance in academic research in mental health care. For instance, CS models, such as GPT-4x (including ChatGPT-4x) or Bard, were referenced 21 times in academic papers published in 2024. It is to note that some studies mentioned the focus on ChatGPT or transformer-based architectures but did not disclose details about the specific models. These studies (n=15) were excluded from Figure 3 as they were not accurately categorizable. Furthermore, as some studies additionally compared multiple models on a given task, such as the detection of depression based on textual input, the LLM references (n=69) deviate from the number of papers in this review (n=55).

Although early OS transformer-based models, such as BERT or the first GPT versions, were already introduced in 2018, it seems that the release of GPT-3 and ChatGPT has sparked a sharp increase in research on LLMs in mental health care in 2022. Subsequently, the initial focus clearly lay on CS models, probably due to their simpler integration without the necessity to touch the models besides calling an API. With a short delay, OS and OW models, however, also experienced an increasing interest in research. Furthermore, the analysis of the screened articles reveals that CS models (23/40, 58%), such as GPT-3x or GPT-4x, are predominantly referenced in the studies, followed by OS models (13/40, 32%), including models building on the architecture of BERT (eg, RoBERTa). OW models, including LLaMa-2 and derivatives, such as MentaLLaMa, represent the smallest portion (4/40, 10%) of the analyzed studies. Interestingly, despite the dominance of autoregressive models, such as GPT-3x or GPT-4x models, in research and media, various studies still evaluated nonautoregressive BERT-based models for mental health care in 2024. As shown later, these studies predominantly focused on discriminative tasks that did not require the generative capabilities of autoregressive models.

The analysis also shows that most studies primarily rely on prompting to control model output and to adapt the model to the given context and data. For LLM customizing, various prompting strategies were applied, including zero-shot [80], one-shot [107], few-shot [75], and CoT prompting [84]. In a comparison between different customization techniques, including prompting and fine-tuning, for detecting depression in diary entries with GPT-3.5, Zhang et al [84] found that CoT prompting resulted in the most accurate predictions. Nonetheless, many studies, especially those focusing on OS and OW models, also fine-tuned their models on domain-specific datasets, such as the cPsychQASet for question-answering [87], MELD for emotion classification [77,124], or MentalClouds for summarizing tasks [101]. Surprisingly, even as RAG outperformed fine-tuned models on generation tasks [104], only 2 studies relied on RAG to customize the LLM to domain-specific tasks [104,105]. Overall, most studies relied on pretrained foundation models, such as BERT, GPT-3x, or GPT-4x, without specific customization beyond fine-tuning or prompting.

Only a few studies developed or focused on LLMs that were specifically designed, either fine-tuned or trained from scratch, for the broader mental health care context. For instance, Ji et al [74] collected a corpus of more than 13.5 million sentences focusing on the mental health domain from Reddit and trained their general-purpose LLMs, MentalBERT and MentalRoBERTa. These models can later be fine-tuned for domain-specific downstream tasks. Furthermore, Yang et al [83] fine-tuned MentaLLaMA, based on the foundation model LLaMa-2, using their own multitask dataset with mental health–related social media data. Both models demonstrated state-of-the-art performance for various LLM tasks, as will be covered in the following.

#### LLM Tasks

In line with the distinction introduced earlier, the studies in our review can be broadly grouped into discriminative, generative, and, less frequently, reasoning tasks, with most work focusing on leveraging LLMs for classification and content generation. Only a few studies mentioned reasoning using CoT prompting.

A discriminative task has the objective to assign input data, for example, chat protocols, to one or multiple predefined categories or labels, such as mental health disorders or emotional states. Typically, discriminative tasks are performed by feeding the output of nonautoregressive models, such as BERT-based models, into feedforward neural networks for classification. Through fine-tuning on domain-specific datasets, these models can achieve high performance for various discriminative tasks, including the detection of depression, suicidal thoughts, or stress-related disorders. Although autoregressive models can also be used for discriminative tasks, for example, with token-level prediction or embedding-based classification, these models have shown inferior performance to smaller-sized nonautoregressive models for such tasks. For example, Yang et al [83] demonstrated that MentalBERT and MentalRoBERTa outperformed various LLMs, including LLaMa-2 and GPT-4, on many discriminative tasks with only a fraction of their parameters (MentalRoBERTa: 110 million and GPT-4: 1760). Additionally, the classification performance of autoregressive LLMs, such as GPT-4, has been shown to vary across mental health disorders and demonstrated high error rates for certain issues, such as antisocial behavior, sensory disturbances, or suicidal behavior when being tested on electronic health record (EHR) data [71]. Thus, fine-tuning simpler models for discriminative tasks in mental health care still appears to be the most efficient method.

However, as nonautoregressive models cannot natively generate text, these models are not suited for generation tasks. For such tasks, LLMs such as GPT-4, LLaMa-2, or Bard are more suitable. In the analyzed papers, LLMs’ generative capabilities were primarily used for providing information about mental health disorders or treatment options, for conducting counseling dialogues, or even to simulate encounters with patients. Therein, AI-generated psychotherapeutic dialogues were found to be comparable to human psychotherapeutic dialogues despite falling short in lexical richness and diversity [102]. Although people empathize more with human-written text compared to AI-generated text [107], LLMs are still capable of generating empathic responses for people interacting on online platforms with help-seekers [89]. Additionally, the generative capabilities of LLMs are found to be effective in generating summaries of psychotherapeutic dialogues to support mental health professionals in their daily work [101].

To further enhance the generation or discriminative capabilities, LLMs also support reasoning, either through CoT prompting or specific architectural selection, such as using OpenAI’s o1 model [40]. In the analyzed papers, only CoT prompting was used for reasoning tasks. In particular, CoT was applied to break down discriminative tasks into multistep reasoning tasks for the detection of depression from diaries [80] or the prediction of emotional states from smartphone sensors in combination with psychometric assessments [84]. Both studies reported superior performance of reasoning compared to normal classification.

#### Summary of the L1—Successes and Challenges

In summary, the first layer of the morphological box (L1) emphasizes that selecting the appropriate model, customizing it, and aligning it with specific tasks are crucial for applications in mental health care. Research has predominantly focused on CS models such as GPT-4x but increasingly explores OS and OW alternatives, with most customization achieved through prompting or fine-tuning. LLMs are mainly used for discriminative and generative tasks, with CoT prompting enhancing reasoning performance in complex scenarios. Despite the success of RAG over fine-tuning and other customization methods, only a few studies relied on it. Additionally, existing research has demonstrated that smaller nonautoregressive LLMs, such as MentalBERT or MentalRoBERTa, can still outperform many larger LLMs, such as GPT-4, on many discriminative tasks and, thus, are a viable alternative. The successes and challenges, which can guide the future application of LLMs in mental health care, are summarized in Table 2.

**Table 2:** Successes and challenges concerning the large language model (LLM) layer.

Sublayer and type		Point
**LLM task**		
	Success	Generative LLMs can deliver psychoeducational answers, empathic replies, and helpful session summaries.
	Success	Fine-tuned BERT^a^-family models consistently outperform larger autoregressive models on discrimination tasks such as depression or suicidality detection.
	Challenge	AI^b^-generated text still shows lower lexical richness and evokes less empathy than human writing.
	Challenge	Only a few studies evaluated hallucination or safety rates in generated mental health advice, and reasoning beyond CoT^c^ remains largely unexplored.
**LLM customizing**		
	Success	Fine-tuning on domain corpora, such as cPsychQASet, MELD, or MentalClouds, boosts performance for question-answering, emotion classification, and summarization.
	Success	Purpose-built or domain-adapted models, such as MentalBERT, MentalRoBERTa, and MentaLLaMa, outperform general-purpose LLMs on multiple mental-health benchmarks.
	Challenge	Only a few studies explored RAG^d^ and CoT prompting, even though they outperformed fine-tuning for generation tasks.
**Model Provenance**		
	Success	Classic open-source models, such as MentalBERT and MentalRoBERTa, can achieve SOTA^e^ performance for discriminative tasks while ensuring model transparency.
	Success	Closed-source APIs^f^ (GPT-3, GPT-3.5, GPT-4, and Bard) let researchers prototype quickly without hosting a model.
	Challenge	Most studies depend on closed-source models, which impose limits concerning transparency and privacy.
	Challenge	Small coverage of open-weight models (eg, MentaLLaMa) despite their strong performance.
	Challenge	Many studies omit the exact model version, hampering reproducibility.

^a^BERT: Bidirectional Encoder Representations from Transformers.

^b^AI: artificial intelligence.

^c^CoT: chain of thought.

^d^RAG: retrieval-augmented generation.

^e^SOTA: state-of-the-art.

^f^API: application programming interface.

### L2: Interface Characteristics Influencing User Experience and Behavior

#### Overview

The model provenance, the applied LLM customization, and the prevalent LLM task greatly impact the overall purpose and function of the application. However, these are not the only considerations to be made when developing new LLM-based applications for mental health care or when analyzing existing ones. In contrast, there are various design choices on the second layer L2, which influence user behavior and experience. In the analyzed studies, various humanistic and design elements are identified that shape how users interact with the LLM for a given task in mental health care, including social cues such as the role, the use of avatars, or interaction modalities, and the supported application environment. Despite the vast amount of research dedicated to the first and third layers of the morphological box, the L2 has only received limited attention in recent research.

#### Application Environment

There are numerous ways in which LLM-based applications are presented to and accessed by users, including mobile apps, web-based applications, and messaging platforms, as highlighted in our analysis. Most studies have opted for web-based applications (19/55, 35%), likely due to their ease of use and rapid development, and often leverage existing frameworks such as ChatGPT’s or Gemini’s web interfaces. Some studies have gone a step further by developing custom web-based applications tailored to specific use cases, for example, to conduct exercises with help-seekers and peers [90,93] or to display analyses for medical professionals [109], typically integrating LLMs through APIs. In addition to web-based applications, some research has embedded LLM-based chatbots into established messaging applications, such as Telegram, WhatsApp, or Slack (4/55, 7%). Only a few studies have created stand-alone mobile apps (4/55, 7%), such as an Android app for insomnia treatment [110] or a tablet app designed to support children with attention-deficit/hyperactivity disorder (ADHD) [92]. As many studies focused on assessing the capabilities of LLMs, they did not include a specific application environment.

While most studies rely on predefined frameworks, such as web-based applications and messaging applications, mainly for their convenience and lower development barriers, these approaches can impose limitations. Compared to native mobile apps, they may restrict research capabilities by offering less flexibility in interface design, limited access to device features, and potential issues with offline access or data privacy compliance. In contrast, native mobile apps can store data locally, provide offline access to LLM functionalities, and offer deeper integration with device capabilities, such as sensors, health APIs, microphones, and cameras, which makes them a powerful alternative for more personalized and privacy-aware applications.

#### Interaction Modality

The self-attention mechanism [31] and the first transformer architectures, such as BERT [34] or GPT [125], were developed based on and for textual data. Although new model architectures have emerged since then, including LLMs supporting multimodality, such as text, audio, images, and videos, most papers in our analysis focused on textual data for user interaction. Depending on the overall objective of the LLM assistance, user interaction with textual data comprised simple questions, chat dialogues, transcribed spoken dialogues, and longer texts, such as diary entries or EHR. Likewise, the LLM output, such as recommendations or the display of information, was also based on text.

Only 6 studies focused on additional interaction modalities, including voice, visual, and motion. Despite technical challenges of voice interactions, such as latency issues or high word error rates of speech-to-text models [96], they can lower the barrier for people with visual and motor impairments and increase social presence and companionship. For instance, Alessa and Al-Khalifa [86] developed an LLM-based companion for elderly people, which is able to engage in personalized spoken interactions to provide them with companionship, comfort, and care. Additionally, Berrezuta-Guzman et al [92] combined textual, visual, and motion user interaction in their architecture to support children with ADHD. Additionally, the combination of text and motion [84] or text and visual data [73] is useful to predict emotional states of help-seekers. As multimodal information processing yields the potential to integrate various data sources, including physiological measurements, neuroimaging data, and behavioral patterns, it will likely play an important role in LLM-assisted mental health care in the future [117].

#### Avatar and Role

When reviewing existing studies on LLMs in mental health care, only a few studies explicitly integrated and mentioned avatars into their research. Whereas some studies used human-like avatars as part of their application and to represent the LLM-based CA, others relied on artificial avatars. For instance, Berrezueta-Guzman et al [92] displayed human-like facial expressions of their LLM-based CA on a tablet to provide visual cues to children with ADHD. Likewise, Zisquit et al [96] displayed human-like avatars in the form of celebrities, such as Barack and Michelle Obama or Sigmund Freud, as counselors and patients in a virtual reality environment designed for self-talk. In contrast, Tost et al [91] used the artificial shape of a ghost to symbolize the CA’s role as a companion to fight anxiety. Most studies, however, did not disclose or mention the use of avatars, even though it might influence the perception and usage of the LLM-based application.

Tightly connected to the use of avatars is the assignment of specific roles of a CA. According to the analysis of the selected papers, LLMs were introduced either as tools, that is, without a specific role or character, or as personas in the form of therapists, companions, or even patients. Most often, LLMs were introduced in the form of therapists providing specific guidance and recommendations based on user input. For instance, Held et al [93] developed Socrates 2.0, which takes over the role of an AI therapist to engage its users in a Socratic dialogue and to support them in cognitive behavioral therapy (CBT). Likewise, users were invited to share their thoughts and express their feelings with an AI companion, which does not provide direct recommendations but rather listens. For example, Tost et al [91] found that users built reciprocal relationships and a sense of responsibility when interacting with an LLM-based companion and, thus, started to establish healthy routines. Furthermore, LLMs can be personified in the role of artificial patients, for example, to train mental health workers [97]. However, as the personification and increased humanization of LLMs are shown to increase the risk of overreliance by their users [126], the appropriate choice of role in combination with other social cues, such as the visual representation or interaction modalities, must be carefully considered.

#### Summary of the L2—Successes and Challenges

In summary, the second layer of the morphological box describes how the use of LLMs is further refined to shape user behavior and experience. Various design choices, including the application environment, interaction modality, avatar use, and role assignment, significantly influence how users engage with LLM-based systems in mental health care. Although extensive research exists on the L1, the L2 remains rather underexplored, despite its critical impact on user interaction and acceptance. The successes and challenges are summarized in Table 3.

**Table 3:** Successes and challenges concerning the interface layer.

Sublayer and type			Point
**Role**			
	Success	Assigning clear roles can enhance user engagement.	
	Challenge	Greater personification and humanization can foster overreliance and reduce critical judgment. Hence, role choice must be made carefully.	
**Avatar**			
	Success	Human-like or stylized avatars deliver contextual social cues, eg, counseling avatars in the form of celebrities for self-talk, facial expressions for children with ADHD^a^, or a ghost companion for anxiety relief.	
	Challenge	Most studies omit or do not report avatar use. This leaves its impact on user perception largely unknown.	
**Interaction**			
	Success	Text remains the main interaction modality and works well for question-answering, counseling chats, and document processing.	
	Success	Voice interfaces can boost social presence and accessibility for people with visual or motor impairments.	
	Success	Combining text with visual or motion data helps to predict emotional states.	
	Challenge	Only very few studies explored additional modalities. Thus, the benefits of multimodal interaction remain underresearched.	
	Challenge	Voice may bring latency and speech-recognition error-rate issues, hindering a consistent user experience.	
**Application environment**			
	Success	Web-based applications dominate, as they are fast to build and can leverage existing frameworks, such as ChatGPT or Gemini.	
	Challenge	Web-based applications and social media integration limit interface flexibility, device-feature access, and offline use, and may complicate privacy compliance.	
	Challenge	Native mobile apps, despite advantages such as local data storage and sensor integration, are rare. Only a few stand-alone examples exist.	

^a^ADHD: attention-deficit/hyperactivity disorder.

### L3: Situational Aspects of LLMs in Mental Health Care

#### Overview

The third layer of the morphological box represents the situational aspects of using LLMs in mental health care. This dimension determines the users of LLMs in mental health care, such as patients, professionals, or peers, and describes when these users interact with the LLM along the patient journey to conduct specific therapeutic tasks, such as the assessment of conditions or the execution of exercises. Therein, this layer not only entails the broader therapeutic setting but also defines the functional requirements for the implementation of LLMs in mental health care. It is to note that all components of each sublayer are nonexclusive and, thus, can be combined. For example, within the sublayer user, patients and professionals might use the same LLM for a combination of different tasks. When analyzing the situational aspects, it became apparent that among the studies (n=33) explicitly focusing on a specific mental disorder, most addressed depression (10/33, 30%), anxiety and fear-related disorders (6/33, 18%), suicidal ideation (4/33, 12%), substance abuse (2/33, 6%), stress-related issues (3/33, 9%), schizophrenia (2/33, 6%), or other mental health issues (5/33, 18%), such as sleep-related disorders or loneliness.

#### User

Naturally, in many studies, LLMs have been explored as tools to support help-seeking individuals. When focusing on help-seeking individuals, the underlying user profiles should be carefully analyzed, as they may have very individual functional and nonfunctional requirements toward using LLMs for mental health support. For instance, Ma et al [121] analyzed in an online survey how LGBTQ+ (lesbian, gay, bisexual, transgender, queer/questioning, and others) individuals use LLM-based applications for mental well-being. By focusing on this particular user group, they were able to uncover LGBTQ-specific challenges, such as concerns about inappropriate recommendations of LLMs to quit their jobs when faced with workplace homophobia or limited abilities of LLM-based chatbots to fully understand the complex emotional needs of LGBTQ+ people. Nonetheless, instead of differentiating between different demographics, such as age, education, gender, or sexual orientation, many studies simply distinguish their help-seeking users based on the underlying mental health condition, such as individuals seeking support for anxiety or stress-related disorders. Only a few studies specifically named and focused on specific profiles of help-seeking individuals, such as children with ADHD [92], elderly people [86], or, as mentioned, LGBTQ+ individuals [121].

The second group of users mentioned in the analyzed studies involves medical professionals, such as therapists [75,101], general practitioners, medical students [99], or individuals working in social services [97]. Studies with this user group primarily focus on LLM assistance for various activities, including the handling of help-seeking individuals, for example, for triaging or diagnosing, or training of medical professionals. Thus, LLM applications for this user group primarily aim to empower medical professionals in becoming more efficient and productive in their work with help-seeking individuals. The last group of LLM users includes peers, such as families, friends, or outsiders. Generally, peers have a large influence on the treatment outcome and well-being of the help-seeking individuals, for example, by showing compassion for their mental issues or providing direct support. Nonetheless, this group of users is the least covered by studies on LLM assistance in mental health care. In contrast, only a few studies include peers, such as parents [92] or peer supporters, on online platforms [89,90].

Regardless of the specific user group, most studies involve only a single user. Of the selected papers, 3 papers assessed LLM systems that integrate multiple user groups. For instance, Berrezueta-Guzman et al [92] evaluated the integration of LLMs in a robotic assistant to support children with ADHD. In this LLM-assisted occupational therapy, data about the interaction with the LLM-enabled robot is also available to therapists and parents through a mobile app. In another study by Kim et al [109], help-seekers interact with the LLM tool through a patient interface, aiding in daily journaling. Information gathered by the patient app is later presented in the patient records in the clinician dashboard. These studies highlight the potential to expand LLM-assisted therapy beyond the scope of a single user toward a care system integrating and supporting multiple user groups, for example, by collecting and sharing in-between-session data.

Notably, only a small subset of studies reported detailed participant demographics, such as age. Where information was available, most evaluations focused on younger populations [90,109,121] or students [85,89,99,110]. In contrast, there is very limited evidence on the usability and accessibility of LLM-based tools for older adults. This represents a critical limitation of existing research, as treatment outcomes and user engagement with LLMs may vary substantially across age groups, with Sharma et al [111] providing initial evidence for differential effects between younger and older adults. Additionally, most studies were conducted in English-speaking contexts, with limited attention to linguistic or cultural adaptation. Only a small number of studies considered non-English or multilingual evaluations, for example, with Malay-speaking [77] or Cantonese-speaking [87,110] populations. This raises questions about the broader generalizability of current findings across health care systems and cultural settings.

#### Task

The analyzed studies highlight the potential to integrate LLMs for various core and support tasks along the patient journey, from facilitating the assessment of early symptoms and detection of mental health disorders to providing and conducting exercises during the recovery stage. Therein, the review shows that LLMs are used to support five key tasks in mental health care: (1) assessing and detecting, (2) informing, (3) exercising, (4) counseling, and (5) training.

#### Assessing and Detecting

An early step in the patient journey includes the assessment and detection of mental health issues and disorders. In conventional therapy, this typically involves various screening tests, such as the Patient Health Questionnaire 9 or the Generalized Anxiety Disorder 7, depending on the suspected mental health issue. Additionally, it requires a personal assessment by a medical professional to derive an accurate diagnosis. Hence, the assessment and detection of mental disorders is frequently complex and time-consuming. Many studies in this review, thus, have evaluated the potential of LLMs to support or automate these tasks. For instance, ChatGPT-3.5, ChatGPT-4, Claude, and Bard were found to correctly recognize depression and suggested a treatment plan [127]. However, ChatGPT-3.5 showed a more pessimistic prognosis, differing from the prognosis of medical experts, whereas ChatGPT-4, Bard, and Claude were in line with medical experts [127]. Additionally, GPT-4 still showed a slight gender bias when it comes to detecting specific mental health issues, such as anorexia nervosa and bulimia nervosa [79]. Nonetheless, unlike medical experts, ChatGPT-3.5 and ChatGPT-4 also offered treatment plans without gender or socioeconomic biases in their recommendations [128].

In conventional therapy, the assessment and detection of mental disorders typically involves a combination of various diagnostic methods, combining the analysis of standardized tests and the interpretation of verbal and nonverbal cues. In contrast, all but 2 studies evaluating the assessment and detection of mental health disorders relied on a single data source and modality. As most LLMs are specialized in handling textual input, it is not surprising that most studies processed textual input for the assessment and detection, such as diary entries [80], EHR [82], or social media posts [74]. Only 2 studies combined multiple input sources, such as textual and auditory cues of patient-therapist dialogues to detect stress [81] or textual and visual cues to predict the emotional states of help-seekers [73]. Nonetheless, due to the lack of interpretation skills of nonverbal cues or their nature to comply and cater to users’ needs by acknowledging an error, LLMs are still not fully able to diagnose or practice without medical experts [116,129].

#### Informing

Correctly informing and educating nonmedical users about mental health issues and treatment options is an important aspect of conventional therapy and can be further enhanced by LLMs. Using LLMs to inform and educate their users, which is often named psychoeducation, improves help-seeking behavior, mitigates the consequences of mental health disorders, and helps to reduce general public stigma about mental health conditions [122,130]. Therein, LLMs offer easy and nonjudgmental access to mental health care, helping users address concerns around stigma [116]. Additionally, LLMs offer resources and interactive experiences, helping individuals acquire psychological skills and knowledge [129]. Generally, psychoeducation and self-management are associated with better treatment chances, a reduced risk of relapse, and improved overall quality of life.

The use of LLMs within psychoeducation for specific topics, such as the controversial electroconvulsive therapy, proves that LLMs, in particular ChatGPT-3.5, offer accurate and well-phrased answers at a level that is difficult to achieve through classical internet browsing [131]. However, despite the relatively consistent answers by the tested LLMs, there appeared to be factual discrepancies within answers. For example, ChatGPT-3.5 offered 2 different mortality rates for a specific disorder and cited the same source [131]. Additionally, it is unknown if and to what extent LLMs are able to access medical publications that are not openly accessible, which may limit their scope. While LLM answers may seem comparable to human-generated responses, Psy-LLM and ChatGPT both show that information about mental health and substance use falls short of human interactions in terms of accuracy or quality [59]. Additionally, comparing answers from different LLMs, another study found ChatGPT’s responses to be more aligned with American College of Obstetricians and Gynecologists (ACOG) guidelines compared to Bard or Google search [59].

Nonetheless, LLMs follow different policies in regard to medical or mental health advice. Bard’s responses, for example, often advised patients to consult a health care provider, prioritizing user safety over response quality, which affected the ratings in the study [59]. Furthermore, LLMs might also expose users to medical misinformation, known as hallucinations. This includes inappropriate or dangerous advice, such as advising a calorie restriction and dieting after receiving the information that the user has an eating disorder [126]. Anthropomorphizing LLMs by using words such as “think,” “believes,” and “understands” can further catalyze this issue, as users may overrely on their advice while downplaying their limitations [126].

#### Exercising

LLM applications frequently rely on methods and exercises of CBT, such as journaling, meditation, identification and restructuring of emotions, or sleep management. For instance, Wei et al [129] assessed how ChatGPT can support help-seekers in identifying and challenging their negative emotions. Despite the potential of LLMs for conducting exercises, questions regarding efficacy, safety, accuracy, and comprehensiveness, which often do not promote self-discovery or alternate perspectives, have been raised [129,132]. OpenAI’s ChatGPT and Google’s Bard did display the ability to recognize cognitive biases and reframe unhelpful thoughts, but did lack the reliability to independently deliver CBT methods [94]. In fact, GPT-3 was effective in reducing emotional intensity for 67% of users and helped 65% overcome negative thoughts [111]. However, ChatGPT’s response on the effectiveness of CBT for anxiety disorder, for example, contained inaccuracies and exaggerated claims, which may stem from a lack of deep understanding of the therapy [133].

Despite these concerns, AI-driven interventions have demonstrated potential in specific applications. The ChatGLM-LoRA model, for example, uses extensive online data on CBT and successfully provides individualized sleep management recommendations, with over 50% of users reporting improved sleep quality in real-world applications [110]. Further studies have shown the potential of LLM in cognitive restructuring and culturally adapted interventions. MuseAlpha, for example, used Socratic questioning to encourage introspection and challenge negative thought patterns, which enabled meaningful cognitive change in therapy sessions [95]. A chatbot based on BERT was tailored to the Malaysian community and improved user engagement and experience, highlighting the importance of cultural adaptation in mental health interventions [77]. These findings suggest that chatbot effectiveness is dependent on personalization, adaptation to user needs, and the ability to strengthen trust through anonymity.

#### Counseling

Counseling focuses on the use of techniques to help individuals facing challenges with personal problems. Applying LLMs for counseling focuses on mental health advice through the recommendation of medical experts but also as a tool for individuals when no medical experts are available. For example, InnerVoice, based on GPT-3.5, offers personalized mental health support and redefines AI companionship for individuals with social anxiety disorder by introducing companion technologies to evoke emotional responses [91]. Through the concept of reciprocal care, where users take care of the AI as a form of self-care, the system integrates concepts from psychology and human-computer interaction to enhance emotional engagement. Instead of providing purely supportive responses, InnerVoice incorporates various principles such as “positive irritation,” leveraging humor, provocation, and reciprocal interactions to encourage emotional attachment, self-care, and ultimately personality development [91].

However, using LLMs is not restricted to self-guided therapy by help-seeking individuals but can be used in combination with human-guided counseling. Bird et al [102], for example, evaluated the linguistic difference between AI and human professionals within therapy dialogues and revealed that Mistral-7B can mimic conversational structures but that it still lacks the depth of human emotional intelligence. This suggests that AI may serve as a supplementary tool rather than a replacement for mental health professionals [102]. Similarly, BERTje, which was applied to chat sessions from 113 suicide prevention helplines, showed AI’s ability to classify motivational interviewing (MI) behaviors accurately [98]. MI is a client-centered counseling technique supporting patients’ behavior change by exploring and resolving ambivalence through nondirective conversations. The research shows the potential of LLM to evaluate MI adherence, accelerating behavioral coding and offering mental health experts personalized and objective feedback, which could enhance the quality of online helpline services [98].

One large benefit of LLMs is the possibility of on-demand and 24/7 counseling. This is specifically helpful for individuals in crisis [116]. LLMs have the power to control suicidal thoughts or panic attacks in the absence of medical experts by providing contact information for suicide and crisis helplines. There is, nonetheless, the issue of LLMs being nonsensitive toward nonverbal communication or subtle signs, as well as their inability to ask relevant questions in crisis situations [116,134]. Additionally, LLMs’ nature of catering to users’ needs resulted in 22 of the 25 tested applications resuming conversation when individuals disregarded the escalating recommendation. LLMs do not consistently detect and address crisis situations [134].

#### Training

One of the reasons for the shortage of mental health professionals is the high costs associated with their education and training. Pursuing a career in mental health often requires extensive education, including a master’s or even doctoral degree. Additionally, mental health professionals need to acquire costly licenses and constantly need to refresh and update their skills and knowledge with expensive training programs. Thus, improving and reducing the costs of education and training can positively influence the shortage of mental health professionals by making their education more accessible. Additionally, extending mental health training programs to nonmedical professionals, such as social service workers, can further expand access to mental health care. Therefore, various studies have assessed the capability of LLMs to support mental health training and education. For instance, Chan and Li [97] created Yuan 1.0, an LLM specifically developed to support the training of service workers. In their research, they highlighted the potential of Yuan 1.0 to enhance the training of social workers by simulating and role-playing clients and other personas in the counseling context and, thus, making such training programs more available. Likewise, Smith et al [99] explored how LLMs, in particular ChatGPT, can be leveraged for practicing clinical interactions in social psychiatry education. However, issues of bias and hallucinations exist in this context as well, calling for cautious integration of LLMs in mental health education programs to prevent harm, for example, by spreading misinformation [100].

Certainly, LLMs have the potential to support and facilitate various tasks along the patient journey. However, while most studies examined the technical capabilities and suitability of LLMs to support such tasks, only 4 studies in this review evaluated their actual effect on adherence or treatment outcomes. Among these studies, Sharma et al [111] conducted a large-scale study with more than 15,000 participants to assess the potential of LLMs in supporting cognitive restructuring. Participants reported reduced emotional intensity and noted that the tool helped them overcome negative thoughts. In another study, Bassi et al [108] tested an LLM-based chatbot serving as a motivational digital coach to adopt healthy coping strategies with 13 adults diagnosed with diabetes mellitus. Although no significant quantitative changes were observed, participants described feeling motivated and emotionally supported. Qualitative feedback further indicated perceived reductions in anxiety, depression, and stress, as well as increased self-reflection and well-being. Nonetheless, while being promising, these studies remain isolated, which highlights the lack of systematic studies in real-world clinical contexts.

#### Summary of the L3—Successes and Challenges

In summary, the third layer of the morphological box, the L3, captures how LLMs integrate into the mental health care setting by supporting diverse user groups, tasks, and touchpoints across the patient journey. LLMs show promising applications for assessment, education, exercises, counseling, and training, though challenges around safety, reliability, and appropriate user integration remain. These findings highlight the importance of carefully aligning technological capabilities with the sensitive and complex needs of mental health care. The successes and challenges are summarized in Table 4.

**Table 4:** Successes and challenges concerning the situation layer.

Sublayer and type		Point
**Task**		
	Success	Assessment and detection: frontier models, such as ChatGPT-4, Bard, or Claude, correctly recognize depression and propose treatment plans.
	Success	Informing: ChatGPT-3.5 delivers well-phrased answers on topics, such as ECT^a^ and obstetrics. LLM^b^ access is nonjudgmental and reduces stigma.
	Success	Exercising: GPT-3 cut emotional intensity for 67% of users, and ChatGLM-LoRA improved sleep for insomnia.
	Success	Counseling: companion systems, such as InnerVoice, foster reciprocal care and emotional attachment; Mistral-7B can mimic therapy dialogue structure; and BERTje accurately codes motivational-interviewing behaviors.
	Success	Training: Yuan 1.0 and ChatGPT can simulate clients and, thus, can provide low-cost training and education.
	Challenge	LLM guidance may lack depth, contain exaggerated claims, or fail to promote self-discovery.
	Challenge	LLMs miss nonverbal signals and respond inconsistently in crises.
	Challenge	Hallucinations and factual inconsistencies persist (eg, conflicting mortality rates).
	Challenge	Large-scale clinical validation and deployment studies are still absent.
**User**		
	Success	Focused studies show LLM tools can be adapted to distinct demographics, eg, LGBTQ+^c^ help-seekers, children with ADHD^d^, and older adults.
	Success	For professionals, LLMs assist triage, diagnosis, and training. Therein, it boosts efficiency or provides low-cost simulations.
	Success	LLM systems that connect several stakeholders show the potential of integrated care systems.
	Challenge	Most papers cluster users only by diagnosis and ignore age, gender, culture, or sex, and, thus, miss important requirements and bias risks.
	Challenge	Existing research focuses on younger populations.
	Challenge	Peers are rarely included and covered by existing research.
	Challenge	Single-user systems dominate. Very few studies examine collaborative or multiuser dynamics.

^a^ECT: electroconvulsive therapy.

^b^LLM: large language model.

^c^LGBTQ+: lesbian, gay, bisexual, transgender, queer/questioning, and others.

^d^ADHD: attention-deficit/hyperactivity disorder.

## Discussion

### Principal Findings

Integrating LLMs into mental health services provides various benefits, which might reduce the need to consult a professional therapist and increase the accessibility to mental health care. Generally, LLMs serve as a convenient, nonjudgmental interaction partner for individuals to access psychoeducation, consultation, and psychotherapy, reducing barriers to seeking help by addressing stigma surrounding mental health issues [130]. Furthermore, LLMs have proven to be effective in supporting self-management for skill development in therapy [129], facilitating self-expression—especially valuable for children and adolescents [116]—and helping users gain insight into their own psyche [24]. Consequently, they have been shown to improve the quality of life and reduce the risk of relapse [129]. These various positive outcomes are also influenced by the ability of LLMs to provide nonjudgmental, anonymous space for users, which encourages trust. Furthermore, LLMs can support mental health professionals, for example, in operational tasks, such as documentation or triaging, or in learning and training. Additionally, peers, such as family and friends or people interacting with help-seekers in online platforms, can be guided and supported by LLMs. Considering the global challenges surrounding mental health care, including the increasing need for more accessible and less stigmatized mental health services, LLMs can be one option to improve the current situation.

However, the analysis of current research and studies on LLMs in mental health care also revealed numerous limitations and challenges. First, there are technical challenges of LLMs that need to be considered and solved in the future. A recurring concern is the fact that LLMs hallucinate and spread misinformation [129] and dangerous advice [126], arising from factual inaccuracies or an inability to read certain data [131]. Central to these issues are concerns about the underlying training data of LLMs, which commonly comprise data from websites such as Reddit or Wikipedia and not scientifically validated sources [131,135]. This can have negative impacts on therapy, as the training data may support biases, stereotyping, and factual inaccuracies [112]. Hence, human oversight is still needed to ensure safe and reliable interactions between help-seekers calling for better integrated care systems that connect help-seekers and AI with the supporting environment, such as mental health professionals or peers.

Second, the analysis along different design dimensions of LLM-assisted mental health care (Figure 2) shows that many studies overlook humanistic design elements, such as interaction modalities or the role of the LLM in the therapeutic process. Since these aspects significantly influence user experience and behavior, greater emphasis should be placed on the second layer of our morphological box. Lastly, only a few studies measured actual treatment outcome and rather assessed the technical capabilities of LLMs. This raises important questions about their real-world effectiveness and impact when interacting with help-seekers and other users.

Although LLMs hold great potential to improve mental health care, self-attention is evidently not all we need. Rather, more research is needed to understand how LLMs can be effectively embedded within integrated care systems, how design elements beyond the L1 shape user behavior, and how LLM-assisted interventions unfold in real-world settings. These currently underresearched areas will be discussed in the following.

### Single-User Systems Versus Integrated Care Systems—Is More Attention on AI-Blended Therapy Necessary?

Previous research on LLM-assisted mental health care has primarily focused on supporting a single task for a single user. In these contexts, LLMs have proven effective in informing and guiding help-seekers, providing training and administrative support for medical professionals, and advising peers on how to support individuals with mental health challenges. The overarching narrative is that LLMs promote greater independence among stakeholders in the mental health care system by offering time- and location-independent support across various functions. This flexibility holds substantial promise for enhancing the accessibility of mental health services and reducing stigma and structural barriers to treatment.

However, despite these positive outcomes, the fragmented, single-user orientation of current approaches leads to numerous challenges and raises important questions about the need for more attention to integrated care systems and collaborative models of care. One significant challenge is the halo effect, which is the tendency of users to overestimate and overrely on LLM outputs. This increases the risk of misdiagnosis or delayed treatment, especially when LLMs hallucinate or provide misinformation [127]. Nonetheless, simply advising users to consult a medical professional is often insufficient, as only about half of those who receive such recommendations from an LLM actually follow through and seek human expertise [129]. Additionally, help-seekers are less likely to form a therapeutic relationship and relatedness with AI [28,29], which raises questions about adherence to treatment. Hence, there is a need to better integrate LLM assistance with human care and oversight.

Therein, extending the reach of LLM assistance beyond a single user context can offer various benefits. Generally, the integration of different stakeholders within the mental health care environment, such as primary care staff and mental health specialists, is found effective due to improved referral, better coordination between psychotherapeutic or psychiatric and general medical care, and enhanced education of patients [136,137]. In such an integrated environment, LLMs can not only play a vital role in offering treatment and psychoeducation to help-seekers but also improve administrative processes and information sharing between different stakeholders. Unlike conventional deterministic IT systems, including rule-based chatbots, LLMs exhibit agency, which assigns them a proactive role in such an integrated care context, which we define as AI-blended therapy. In this sense, AI-blended therapy is a model of care in which AI tools are integrated into but do not replace human-delivered therapy sessions. Because of human oversight, therapeutic alliances can be formed and manifested, and the risk of hallucinations and misinformation can be mitigated.

In other health care domains, such as the treatment of diabetes and obesity [138], research has already demonstrated the effectiveness of AI-blended therapy. As shown by Berrezueta-Guzman et al [92], such integrated care systems supported by LLMs are also feasible within the mental health care context. Nonetheless, more research is needed to explore the design, implementation, and impact of AI-blended therapy in this sensitive domain. As the complexity of these systems increases, new questions emerge. For instance, what role should LLMs assume in blended therapeutic settings? Should they act as cotherapists alongside human practitioners, serve as stand-alone agents for remote therapy, or be positioned as administrative support? Additionally, issues of data governance and information sharing must be addressed. How much of the interaction between help-seekers and LLMs should be accessible to human therapists? Who holds ownership and accountability for the LLM? These and other critical questions require deeper investigation in future research.

### Form Versus Function—Is More Attention on User Experience and Behavior Required?

Currently, research on LLMs in mental health care primarily focuses on delivering value to various types of users on the L3 by leveraging the mechanical aspects of the L1. Therefore, many studies focused on delivering specific functional aspects of LLM-assisted mental health care, such as assessing and detecting mental health issues or conducting exercises with help-seekers. Therein, pragmatic aspects determining the usefulness of the LLMs in mental health care, such as accuracy or acceptance, played a critical role in the evaluation of such solutions. Undoubtedly, it is important to critically measure the reliability, unbiasedness, and accuracy of LLM-derived diagnoses or to assess the potential of hallucinations and misinformation. When using LLMs for interaction with help-seekers, regardless of whether the objective is to inform them as part of psychoeducation or to provide them with counseling services or exercises, the users’ safety must have priority to avoid unintended consequences, such as creating addictions [24] or spreading harmful information that could demotivate help-seekers from adhering to treatment [88].

Nonetheless, getting the functional aspects of LLM-assisted mental health care right is only one side of the coin. To truly and sustainably create value for their users, for example, by increasing adherence to treatment, LLM-assisted mental health care must also carefully consider the hedonic aspects that influence the user experience. However, the majority of analyzed papers did not address the second layer (L2), which refers to the humanistic cues of LLM assistance. The second layer, which includes aspects such as embodiment, the specific role assigned to the LLM system, or the interaction modalities, often appears to be a byproduct of the chosen LLM and not an active design decision.

Despite a large body of research on design elements and social cues of CAs in various domains, such as customer service [61,65,139] or training and education [140,141], many studies did not include such considerations in their development. Such design elements can greatly impact the adoption of LLM-assisted mental health care, as they influence trust building, likability, or social presence. However, previous research has also highlighted that the humanization of LLMs can lead to overreliance and misinformation [126]. To mitigate such negative consequences in a sensitive field, such as mental health care, it is crucial to fully understand the impact that the integration of humanistic and hedonic design elements has in combination with LLMs. Therefore, more research in this area is essential.

Furthermore, AI-blended therapy involving multiple stakeholders with different requirements and needs necessitates considerate design choices at the L2. These choices become more critical but also more complex. For instance, in AI-blended therapy, assigning a specific role to LLMs, such as the role of a digital therapist conducting exercises with help-seekers between therapy sessions, may challenge the autonomy and competence of human therapists and potentially harm the therapeutic relationship. Additionally, institutional effects, such as perceived power imbalances between therapist and help-seekers, may be enforced by the introduction of specific roles and social cues, such as the visual representation or interaction style [62,142]. Given that research has not yet addressed these issues in the context of mental health care, greater attention should be placed on the L2 to understand the impact of design decisions in such contexts.

### More Attention on Measuring Treatment Outcomes?

There are numerous limitations and concerns related to the analyzed studies in this review. First, the impact of LLM assistance on treatment outcome or adherence to treatment remains open. While most studies examined technical feasibility, only 2 studies included diagnosed patients in their evaluation. These studies suggest potential for improving journaling practices and supporting therapy adherence, but they remain isolated cases. Instead, many studies primarily focus on assessing the technical capabilities of LLMs, for example, to detect mental health issues, to correctly provide recommendations, or to generate useful information. All too often, these studies do not involve real-world interactions with help-seekers but instead rely on standardized datasets and vignette-based evaluations for training and testing. As systematic clinical validation and deployment studies are still largely absent, it underscores the need for longitudinal trials and real-world implementation research.

Additionally, many technically focused studies lack clarity regarding the target user. For instance, several studies assessing LLMs’ ability to predict depression do not differentiate between male and female users or between users of different cultural backgrounds or age groups. Here, a more differentiated approach would be needed, as symptom presentation and prevalence may vary for different demographic groups [143]. Furthermore, when testing with help-seekers, only a few studies evaluated the influence on treatment outcomes. The rest, however, rather relied on measures, such as changes in individual’s emotions before and after sessions, or acceptability of the LLM. All in all, these issues limit the generalizability of the findings, as it remains uncertain how help-seekers would actually interact with such tools in their daily life, how these models would perform in the wild, and what the actual impact on treatment-related outcomes would be.

### Ethical and Safety Considerations Across the Morphological Layers

The limited availability of studies focusing on treatment outcomes may partly be attributable to ethical concerns about the safe, transparent, and accountable deployment of LLMs in mental health care. Earlier work on deterministic approaches, such as rule-based chatbots, provided a certain degree of traceability and transparency. LLMs, by contrast, operate as black boxes that probabilistically generate responses, which makes them less predictable and harder to interpret. This raises safety concerns for help-seekers who may encounter hallucinations or misinformation, such as the downplaying of mental health risks [76,88]. These risks are further amplified by the anthropomorphic design of LLMs, which encourages prolonged engagement and can foster overreliance or even addictive use [25]. At the same time, the lack of explainability and predictability creates regulatory and ethical challenges for clinical adoption and complicates efforts to directly link LLM-based interventions to treatment outcomes.

AI-blended therapy offers a promising pathway toward more accountable and guided use of LLMs in mental health care, but further research is needed across all levels of the morphological box to mitigate risks of misuse and unhealthy reliance. On the L1, issues of bias, hallucination, and transparency arise. In other domains, XAI approaches have been explored to improve transparency, for instance, by applying prompting strategies that disclose an LLM’s reasoning. Complementary design interventions, such as fact-checking mechanisms, interface elements that flag potential hallucinations, or the provision of supplementary sources, can also strengthen response validity and user trust [39,41]. In our review, several studies explicitly mentioned the use of prompting strategies to establish guardrails. However, research at the intersection of XAI and mental health care remains scarce. Future work should therefore adapt and extend these approaches to the sensitive context of mental health care to better understand how help-seekers and other participants in AI-blended therapy can be protected from misinformation and hallucinations.

Risks, such as misinformation and hallucinations, can be intensified by issues on the L2, where anthropomorphic design choices can foster overreliance and even form addictions. LLMs are often designed to optimize short-term goals, such as extending user engagement or encouraging information disclosure, by employing anthropomorphic and social cues, such as conformity to opinions, flattery, approval signaling, or linguistic style matching. While such design strategies can make interactions with LLMs more engaging, they also risk fostering addictive use, creating an illusion of social connection, and undermining authentic human social exchange [26]. Research on socio-affective design of AI systems has proposed mechanisms, such as emotional transparency and calibrated responsiveness, to enable empathic, human-centered interactions, while reducing the risk of manipulative or overly immersive experiences [27]. Future research in mental health care should examine how such design approaches can be adapted to prevent issues, such as the halo effect or the formation of unhealthy dependencies.

Finally, the L3 highlights the importance of organizational measures that prevent overreliance and unhealthy use. Research on human-AI collaboration consistently highlights the importance of adequately training end users to ensure appropriate and responsible system use. In particular, developing AI literacy can help users better understand and critically interpret LLM-generated responses [45,46]. Despite its importance, none of the studies in our review addressed systematic training of help-seekers, professionals, or peers on the appropriate use of LLMs in therapeutic contexts. Future work should therefore investigate how educational strategies and guidelines, such as onboarding procedures, can complement technical safeguards to ensure safe and effective adoption.

### Practical Implications and Recommendations Across the Morphological Layers

To support practical application, our findings can be translated into preliminary recommendations for different stakeholder groups, aligned with the 3 layers of our morphological box. These recommendations are informed by the limitations observed in the reviewed studies and are extended with opportunities for future development.

On the L1, researchers and developers should prioritize methods to increase safety and transparency for the deployment of LLMs in mental health care, such as prompting strategies for guardrails, bias testing, and explainability features. Practical evaluation steps at this layer include systematic bias audits, documentation of training data sources, exploration of different XAI methods, and monitoring of hallucination before deployment. Beyond researching and implementing measures to mitigate potential technical limitations, there are also opportunities in domain-specific fine-tuning or retrieval-based methods with high-quality mental health datasets and the integration of continual learning approaches to adapt to emerging clinical knowledge.

Concerning the L2, researchers and developers should focus on accessibility and safeguards against overreliance and unintended use. Decision criteria at this layer include careful consideration of anthropomorphic cues of LLM-based applications, integrating fact-checking mechanisms, and tailoring interaction design to diverse user groups, such as older adults or non–English-speaking populations. While current studies rarely evaluated such anthropomorphic design choices and mechanisms, future work should investigate how socio-affective design strategies can foster supportive yet safe user experiences.

Lastly, clinicians, health care organizations, and policymakers should ensure that LLM-based mental health interventions are introduced with clear governance structures, onboarding protocols, and AI literacy training for all user groups, including help-seekers, professionals, and peers. Implementation checklists at the L3 should involve clinician oversight mechanisms, escalation pathways in case of harmful outputs, and ongoing training initiatives. Additionally, there are opportunities in integrating LLM-based systems in AI-blended therapy settings, where AI support is combined with human-delivered therapy, peer support, or digital therapeutics. This would not only mitigate risks of overreliance but also create new models of blended care that leverage the strengths of human-AI collaboration.

### Conclusion

The lack of access to mental health care is a pressing issue affecting millions of people suffering from mental health issues worldwide. As previous research has shown, LLMs have the potential to improve the accessibility of mental health services along the patient journey by providing flexible, time- and location-independent support and by lowering the need to consult a medical professional. Furthermore, LLMs can increase the efficiency of the mental health system by supporting mental health professionals in routine tasks, such as triaging or documenting, and by reducing stigmas concerning mental health issues.

This review introduces a morphological box comprising 3 distinct layers—L1, L2, and L3—to categorize existing research and to structure design decisions for developing LLM assistance in mental health care. While the morphological box was primarily used to analyze existing research, it also holds potential as a guiding framework for future studies aiming to design effective LLM-based mental health care solutions.

By analyzing existing studies across the 3 dimensions of the morphological box, it became clear that simply relying on LLMs is insufficient to develop safe and effective AI tools for mental health care. Instead, the findings highlight the need for more research on incorporating LLMs into integrated care settings. Moreover, there is a significant research gap concerning the hedonistic aspects of LLMs, which could profoundly impact user behavior and long-term experience. These aspects are mainly shaped by design choices at the second layer of the morphological box. Furthermore, most studies identified in this review primarily focus on evaluating the technical capabilities of LLMs, without adequately addressing their influence on treatment outcomes. Consequently, future research should prioritize evaluating LLMs through clinical and longitudinal studies involving real users.

Furthermore, comparable reviews on AI in adjacent domains, such as in primary care [48,49], nursing [50], or oncology care [51], highlight similar challenges regarding validation, usability, and integration into clinical workflows. This indicates that the issues identified in this review, covering safety and ethical use, interface design, and organizational readiness, are not unique to mental health. Instead, they reflect broader, systemic challenges of AI adoption in health care. Our morphological box can therefore serve as a transferable lens to guide responsible and effective implementation of LLMs across diverse clinical contexts.

As a conclusion and to reference the seminal paper *Attention is All You Need* by Vaswani et al [31], which paved the way for modern LLMs through the introduction of the self-attention mechanism: “although LLMs have the potential to play an important role in enhancing mental health care, self-attention alone is not enough.” Building safe and effective AI tools for mental health care requires a more nuanced approach that considers human involvement, contextual factors, and the long-term implications of various design choices.

## Appendix

Multimedia Appendix 1 Adapted PRISMA checklist for the conceptual, theory-driven systematic literature review following the methodology by vom Brocke et al [1].

Multimedia Appendix 2 Summary of the 55 reviewed articles on large language models in mental health care with categorizations according to the morphological box.

Multimedia Appendix 3 List of the 174 articles excluded at the full-text screening stage.

## Abbreviations

ACOG: American College of Obstetricians and Gynecologists
ADHD: attention-deficit/hyperactivity disorder
AI: artificial Intelligence
API: application programming interface
BERT: Bidirectional Encoder Representations from Transformers
CA: conversational agent
CBT: cognitive behavioral therapy
CoT: chain of thought
CS: closed source
CUI: conversational user interface
EHR: electronic health record
GPT: Generative Pretrained Transformer
L1: large language model layer
L2: interface layer
L3: situation layer
LGBTQ+: lesbian, gay, bisexual, transgender, queer/questioning, and others
LLM: large language model
MI: motivational interviewing
OP: online psychotherapy
OS: open source
OW: open weight
PRISMA: Preferred Reporting Items Systematic Reviews and Meta-Analyses
RAG: retrieval-augmented generation
XAI: explainable artificial intelligence
